# AMSTAR 2: a critical appraisal tool for systematic reviews that include randomised or non-randomised studies of healthcare interventions, or both

**DOI:** 10.1136/bmj.j4008

**Published:** 2017-09-21

**Authors:** Beverley J Shea, Barnaby C Reeves, George Wells, Micere Thuku, Candyce Hamel, Julian Moran, David Moher, Peter Tugwell, Vivian Welch, Elizabeth Kristjansson, David A Henry

**Affiliations:** 1 Ottawa Hospital Research Institute, Clinical Epidemiology Program, Ottawa, Canada; 2 Bruyère Research Institute, Ottawa, Canada; 3 School of Epidemiology and Public Health, Faculty of Medicine, University of Ottawa, Ottawa, Canada; 4 School of Clinical Sciences, University of Bristol, Bristol, UK; 5 University of Ottawa Heart Institute, Ottawa, Canada; 6 The Hospital for Sick Children, the Genetics and Genome Biology Program, Toronto, Canada; 7 Department of Medicine, The Ottawa Hospital, Ottawa, Canada; 8 Centre for Research in Educational and Community Services, School of Psychology, Faculty of Social Sciences, University of Ottawa, Canada; 9 Centre for Research in Evidence-Based Practice, Bond University, Gold Coast, Australia;; 10 Dalla Lana School of Public Health, University of Toronto, Toronto, Canada; 11 Institute for Clinical Evaluative Sciences, Toronto, Canada

## Abstract

The number of published systematic reviews of studies of healthcare interventions has increased rapidly and these are used extensively for clinical and policy decisions. Systematic reviews are subject to a range of biases and increasingly include non-randomised studies of interventions. It is important that users can distinguish high quality reviews. Many instruments have been designed to evaluate different aspects of reviews, but there are few comprehensive critical appraisal instruments. AMSTAR was developed to evaluate systematic reviews of randomised trials. In this paper, we report on the updating of AMSTAR and its adaptation to enable more detailed assessment of systematic reviews that include randomised or non-randomised studies of healthcare interventions, or both. With moves to base more decisions on real world observational evidence we believe that AMSTAR 2 will assist decision makers in the identification of high quality systematic reviews, including those based on non-randomised studies of healthcare interventions*.*

**Summary points**

Systematic reviews of studies of healthcare interventions effects often include non-randomised studies
AMSTAR is a popular instrument for critically appraising systematic reviews of randomised controlled clinical trials
AMSTAR underwent further development to enable appraisal of systematic reviews of randomised and non-randomised studies of healthcare interventions
The revised instrument (AMSTAR 2) retains 10 of the original domains, has 16 items in total (compared with 11 in the original), has simpler response categories than the original AMSTAR, includes a more comprehensive user guide, and has an overall rating based on weaknesses in critical domains
AMSTAR 2 is not intended to generate an overall score
With moves to base more decisions on real world observational evidence, AMSTAR 2 should assist in the identification of high quality systematic reviews

With the rapid increase in biomedical publishing, keeping up with primary research has become almost impossible for healthcare practitioners and policy makers.^1^ Consequently, healthcare decision makers rely on systematic reviews as one of the key tools for achieving evidence based healthcare.^2^ Systematic reviews provide an opportunity to base decisions on accurate, succinct, credible, and comprehensive summaries of the best available evidence on a topic.^2^

Uncritically accepting the results of a single systematic review has risks. One of us (DM) led efforts to improve standards for reporting of systematic reviews, which led to the PRISMA (Preferred Reporting Items for Systematic Reviews and Meta-Analyses) statement.^3^ The reporting guide for systematic reviews of observational (non-randomised) studies is MOOSE (Meta-analysis of Observational Studies in Epidemiology).^4^ The quality of reporting of a systematic review may, however, more accurately reflect authors’ ability to write in a comprehensible manner rather than the way they conducted their review. This underscores the need for guidelines that evaluate the way in which reviews are planned and conducted.^5^ ^6^

The Cochrane Collaboration Handbook provides a comprehensive guide for review authors, but it does not provide a concise critical appraisal instrument for completed reviews.^5^ Several instruments have been designed to evaluate individual studies that are being included in systematic reviews or how certain steps (eg, meta-analysis, testing for publication bias) should be conducted.^7^ ^8^ ^9^ ^10^ ^11^ ^12^ ^13^ ^14^ ^15^ But relatively few instruments assess all important steps in the conduct of a review.^16^ ^17^ ^18^ ^19^ ^20^ ^21^

AMSTAR (A MeaSurement Tool to Assess systematic Reviews), published in 2007, is one of the most widely used instruments.^22^ ^23^ ^24^ AMSTAR was designed by us and our colleagues as a practical critical appraisal tool for use by health professionals and policy makers who do not necessarily have advanced training in epidemiology, to enable them to carry out rapid and reproducible assessments of the quality of conduct of systematic reviews of randomised controlled trials of interventions. Since publication, several critiques of the instrument have been published.^25^ ^26^ ^27^ ^28^ ^29^ ^30^ ^31^ These critiques plus feedback received at workshops and developments in the science of systematic reviews pointed to a need to revise and update the original AMSTAR instrument.

## Inclusion of non-randomised studies in systematic reviews

Almost half of published systematic reviews now include non-randomised studies of intervention effects.^4^ ^32^ ^33^ ^34^ There are many concerns about the conduct and reporting of systematic reviews of non-randomised studies.^32^ ^35^ ^36^ To summarise, non-randomised studies of healthcare interventions (an important focus of this revision of AMSTAR) are subject to a range of biases that are either not present or are less noticeable in randomised controlled trials, thus requiring different risk of bias assessments. Observational studies are increasingly conducted within large population databases, sometimes with hundreds of thousands or even millions of recipients of healthcare interventions. These generate precise estimates of intervention effects, which may be inaccurate because of residual biases. If these estimates are combined with those from the (generally smaller) randomised controlled trials, the meta-estimates will be weighted towards the observational study estimates. The original AMSTAR instrument did not include an assessment of the risk of bias in non-randomised studies included in a review, which is a key issue given the diversity of designs that such studies may use and the biases that may affect them.

## Development of AMSTAR 2

The development and validation of the original AMSTAR instrument (published in 2007) has been described in detail elsewhere.^22^ ^23^ ^24^ Briefly, the original list of items was created from the results of a scoping review of the then available rating instruments. This review identified many over-lapping appraisal items, mainly from two extensively cited reports.^16^ ^17^ The lists of items from these reports were combined and reduced by factor analysis. After pilot testing, items were reworded as needed and the reliability and usability of the tool was assessed. A modified version was validated externally and performed well against the global judgments of a panel of content experts.^23^ The publications describing the original AMSTAR instrument were widely cited and the instrument has been used and critiqued extensively.^22^ ^23^ ^24^ ^25^ ^26^ ^27^ ^28^ ^29^ ^30^ ^31^

We convened an expert group, comprising authors of the original instrument, members with expertise in the conduct of non-randomised studies, development of appraisal instruments, biostatistics, and study designs. The expert group met for a day in Ottawa, Canada and members were presented with the results of updated literature reviews on relevant critical appraisal instruments, the results of surveys of AMSTAR users, recorded experience of participants in AMSTAR workshops at Cochrane Colloquiums in 2015 and 2016, feedback from the AMSTAR website (www.amstar.ca), and published critiques of the original instrument.^16^ ^17^ ^18^ ^19^ ^20^ ^21^ ^22^ ^23^ ^24^ ^25^ ^26^ The perspective adopted by the expert group was to increase the value of AMSTAR as a broad critical appraisal instrument designed primarily for systematic reviews of studies of healthcare interventions. The expert group considered that revisions should address all aspects of the conduct of a systematic review, and the challenges of including non-randomised studies. They also thought the revised instrument should function as a teaching aid and as a concise checklist for those conducting reviews. The revisions were not intended to deal with the special requirements of diagnostic test reviews, individual patient data meta-analyses or network meta-analyses, scoping reviews, or realist reviews.^37^ ^38^ ^39^ ^40^ ^41^

We used a nominal group technique to propose and then prioritise specific changes to the instrument and to agree on the draft wording of items. Based on their experience of the instrument and the presentations made at the meeting, participants were asked to record their ideas independently and privately. The ideas were then enunciated in a round-robin format. One idea was collected from everyone, in turn, and presented to the group by the facilitator. This process was continued until all ideas had been listed. Individuals then privately recorded their judgments and rankings. These were aggregated statistically to derive the group judgments. The following changes were agreed on (these are not listed in order of priority as all were considered important enough to mandate modifications to the instrument):

- Simplify the response categories
- Align the definition of research questions with the PICO (population, intervention, control group, outcome) framework
- Seek justification for the review authors’ selection of different study designs (randomised and non-randomised) for inclusion in systematic reviews
- Seek more details on reasons for exclusion of studies from the review
- Determine whether the review authors had made a sufficiently detailed assessment of risk of bias for the included studies (whether randomised or non-randomised)
- Determine whether risk of bias with included studies was considered adequately during statistical pooling of results (if this was performed)
- Determine whether risk of bias with included studies was considered adequately when interpreting and discussing the review findings.

A description was formulated for each of the draft items. A small subgroup refined the wording of the items and assembled the draft instrument for testing. Initial pilot testing was performed by group members. Draft versions were presented at workshops held at the Cochrane Colloquiums in 2015 and 2016, where feedback directed further modifications and redrafting of the instrument. The version of the instrument presented here was subject to inter-rater reliability and usability testing.

## Comparison with the original instrument

The supplementary figure provides details of the new instrument (AMSTAR 2). Ten domains were retained from the original tool, with changes to the wording of items based on feedback about the original instrument and experience of testing drafts of the new instrument. Two domains were given more detailed coverage in AMSTAR 2 than in the original instrument: duplicate study selection and data extraction now have their own items (they were combined in the original tool). The possible influence of funding sources is now considered separately for individual studies included in the review and for the review itself. Previously they were combined in one item. We added more detailed and separate considerations of risk of bias for randomised and non-randomised studies. Both sub-items are based on content from the Cochrane risk of bias instruments for randomised and non-randomised (ROBINS-I) studies.^42^ ^43^ One domain was removed—consideration of grey literature, previously a separate item, is now handled in the item on literature searching.

In total, four domains were added. Two of these came directly from the ROBINS-I tool—namely, elaboration of the PICO and the way in which risk of bias was handled during evidence synthesis.^43^ One of the other new domains—discussion of possible causes and significance of heterogeneity—is an elaboration of content in the original AMSTAR tool. Another new domain—justification of selection of study designs—was part of the adaptation of AMSTAR to deal with non-randomised designs.

The domain specific questions in AMSTAR 2 are framed so that a “Yes” answer denotes a positive result. We removed the “not applicable” and “cannot answer” options in the original AMSTAR instrument because we believe that all domains are relevant to contemporary systematic reviews of healthcare interventions. If no information is provided to rate an item, the review authors should not be given the benefit of doubt and the item should be rated as a “No.” We have provided a “partial Yes” response in some instances where we considered it worthwhile to identify partial adherence to the standard.

## Rationale for selection of items

Here we summarise our thinking behind the items in AMSTAR 2, which are numbered as in the instrument (see supplementary figure). Supplementary appendix 1 provides a more complete user’s guide.

*1. Did the research questions and inclusion criteria for the review include the components of PICO?*

It is common practice to use the PICO description (population, intervention, control group, and outcome) as a convenient and easily memorised framework for a study question. Sometimes a timeframe should be added if this is critical in determining the likelihood of a study capturing relevant clinical outcomes (eg, an effect of the intervention is only expected after several years).

2. *Did the report of the review contain an explicit statement that the review methods were established prior to the conduct of the review and did the report justify any significant* *deviations from the protocol?*

Systematic reviews are a form of observational research, and the methods for the review should be agreed on before the review commences. Adherence to a well developed protocol reduces the risk of bias in the review. Authors should show that they worked with a written protocol with independent verification.

3. *Did the review authors explain their selection of the study designs for inclusion in the review?*

For some questions, for instance the effects of policy changes, or for ethical reasons, non-randomised studies may be the only studies addressing the review question. With an expansion of AMSTAR 2 to appraise reviews that include randomised controlled trials or non-randomised studies, or both, it is important that authors justify the inclusion of different study designs in systematic reviews. The authors should indicate that they followed a strategy. When both randomised and non-randomised studies address the same question about the effects of an intervention, we believe that authors should consider whether a review that is restricted to randomised controlled trials will give an incomplete summary of the important effects of a treatment.

*4. Did the review authors use a comprehensive literature search strategy?*

The importance of adequate literature searching in systematic reviews is well established.^5^ This item was carried over with minimal changes to the wording from the original instrument. We have made the response options clearer in AMSTAR 2 and provide more detailed guidance on completion of the item, particularly in relation to the identification of non-randomised studies (see supplementary appendix 1).

*5. Did the review authors perform study selection in duplicate?*

Best practice requires two review authors to determine eligibility of studies for inclusion in systematic reviews.^5^ This involves checking the characteristics of a study against the elements of the research question. In the original AMSTAR, this item covered determining both study eligibility and data extraction. The expert group believed that they were sufficiently distinct processes to merit separate items in AMSTAR 2.

*6. Did the review authors perform data extraction in duplicate?*

The expert group recognised that data extraction might be more complex for non-randomised studies of healthcare interventions as it usually involves extraction of measures of treatment effects and other associations that have been adjusted for potential confounding, rather than raw outcome data from treated and control groups. A study report may present multiple treatment effects; judgment is therefore needed to select the one that conforms best to the PICO question and is at lowest risk from confounding.

*7. Did the review authors provide a list of excluded studies and justify the exclusions?*

In the revised instrument we consider excluded and included studies separately. Excluded studies should be accounted for fully by review authors, otherwise there is a risk that they remain invisible and the impact of their exclusion from the review is unknown.

*8. Did the review authors describe the included studies in adequate detail?*

The revised instrument requires review authors to provide detail about research designs, study populations, interventions, comparators, and outcomes. The detail should be sufficient for appraisers to make a judgment about the extent to which the studies were appropriately chosen (in relation to the PICO) and whether the study populations and interventions were relevant to their questions. This information is needed to determine the extent to which the results of different studies should be combined, help explain heterogeneity, and assist those applying the results.

*9. Did the review authors use a satisfactory technique for assessing the risk of bias (RoB) in individual studies that were included in the review?*

Biases can be introduced at several stages in the design, planning, conduct, and analysis of a study. This item replaces a less detailed item on “scientific quality.” The item specifies domains of bias for randomised and non-randomised studies that should have been considered by reviewers, based on the relevant Cochrane instruments.^42^ ^43^ In AMSTAR 2 we ask whether the review authors made an adequate assessment of study level efforts to avoid, control, or adjust for baseline confounding, selection biases, bias in measurement of exposures and outcomes, and selective reporting of analyses or outcomes, or both. The guidance document (see supplementary appendix 1) and the ROBINS-I report provide more detail.^43^ We decided not to include assessment of time varying confounding, performance biases, and biases due to missing data, although they are currently included in ROBINS-I.^43^ This was because of the complex nature of techniques used to adjust for these potential sources of bias and the frequent lack of data (in contemporary primary studies) to enable assessment of these items. Version 2.0 of the Cochrane risk of bias instrument for randomised controlled trials is now available in draft form, and AMSTAR 2 will be aligned with this in the future.^44^

*10. Did the review authors report on the sources of funding for the studies included in the review?*

We added a consideration of funding sources in the light of evidence from several sources that the results of industry funded studies sometimes favoured sponsored products, and that industry funded studies were less likely to be published than those that were independently funded.^45^ ^46^ ^47^ Such influences may not be detected as flaws in design or methods (item 9).

*11. If meta-analysis was performed, did the review authors use appropriate methods for statistical combination of results?*

This is a modified version of an item in the original instrument and is judged separately for randomised and non-randomised studies. Review authors should have stated explicitly in the review protocol the principles on which they based their decision to perform meta-analysis of data from the included studies. This includes the extent to which the studies are compatible (in terms of patients, controls, and interventions) and the value of a single pooled effect (for instance from several compatible but underpowered studies). Where reviewers consider it appropriate to conduct a meta-analysis, the inclusion of non-randomised studies increases the complexity of the analyses and may increase heterogeneity (see supplementary appendix 1).

*12. If meta-analysis was performed, did the review authors assess the potential impact of RoB in individual studies on the results of the meta-analysis or other evidence synthesis?*

This is a new item that requires reviewers to examine how results vary with inclusion or exclusion of primary studies judged to be at high risk of bias. In cases where review authors have chosen to include only high quality randomised controlled trials there may be little discussion of the potential impact of bias on the results. But where they have included randomised controlled trials of variable quality or non-randomised studies they should assess the impact of study level risk of bias on the results of the review.^48^

*13. Did the review authors account for RoB in primary studies when interpreting/discussing the results of the review?*

This is a modification of an item from the original instrument. With a greater emphasis on assessing risk of bias, the expectation is that reviewers will make explicit reference to the potential impacts of risk of bias when interpreting and discussing the results of their review and in drawing conclusions or making recommendations.

*14. Did the review authors provide a satisfactory explanation for, and discussion of, any heterogeneity observed in the results of the review?*

This item is carried over with modified wording from the original instrument. It is important that reviewers investigate possible causes of heterogeneity, including variation in those elements included in the PICO framework (see item 1) and those arising from design and methodological considerations (see item 9). With the inclusion of non-randomised studies, variations in design and analysis may contribute to heterogeneity.

*15. If they performed quantitative synthesis did the review authors carry out an adequate investigation of publication bias (small study bias) and discuss its likely impact on the results of the review?*

This item is carried over from the original instrument but with modified wording. Publication bias is an important problem but it can be difficult for authors to resolve completely. Typically, statistical tests (several are available) or graphical displays are used and if the results are positive they indicate the presence of publication bias. Negative test results are not a guarantee of the absence of publication bias as they are insensitive. A minimum of 10 studies are required to show funnel plot asymmetry.^5^ The underlying tendency to selectively publish small positive studies may be compounded by the effects of lower methodological quality of small studies, a greater tendency to selectively report results, and increased clinical heterogeneity when conducted in patient subgroups.^49^

*16. Did the review authors report any potential sources of conflict of interest, including any funding they received for conducting the review?*

This item is carried over with modified wording from the original instrument and is now separate from consideration of funding of the primary studies included in the review (item 10). As with primary studies, review authors should report their funding sources.^50^ ^51^

### Identification of critical domains

All steps in the conduct of a systematic review and meta-analysis are important, but we believe that seven domains can critically affect the validity of a review and its conclusions (box 1). Two of these concern risk of bias, whether it has been assessed adequately and how it can influence the results of a review. The prominence we give to risk of bias is because AMSTAR 2 is going to be used to appraise many systematic reviews that include non-randomised studies.

**Box 1 AMSTAR 2 critical domains**

- Protocol registered before commencement of the review (item 2)
- Adequacy of the literature search (item 4)
- Justification for excluding individual studies (item 7)
- Risk of bias from individual studies being included in the review (item 9)
- Appropriateness of meta-analytical methods (item 11)
- Consideration of risk of bias when interpreting the results of the review (item 13)
- Assessment of presence and likely impact of publication bias (item 15)

We recognise that the items listed in box 1 will not always be regarded as critical; for example, risk of bias related items may be considered less important when a review is confined to high quality randomised controlled trials. Other circumstances where the critical nature of items may be questioned are when a review team are using meta-analysis to summarise a known literature base (eg, the output from one or more established clinical trial collaborative groups). In this circumstance the adequacy of the literature search (item 4), listing of excluded studies (item 7), and possibility of publication bias (item 15) may not be considered critical. If a meta-analysis was not performed, the item covering the appropriateness of the meta-analytical methods (item 11) will not apply. However, it is important in this circumstance that appraisers are alert to the possible impact of risk of bias when review authors select individual studies to highlight in a narrative summary.

Flaws in the items that we have identified as critical may not be fatal if further information (eg, directly from the review authors) indicates that the original response option was wrong. This may provide reassurance about the review findings or enable an amendment of the review through additional analyses. We emphasise that our listing is a suggestion and appraisers may add or substitute other critical domains. For example, the failure to include non-randomised studies (item 3) in a review of adverse outcomes of treatment may be a critical flaw, as would the inability to explain large variations in treatment effects across a body of studies (item 14).

### Applying AMSTAR 2 to systematic reviews

If one or more systematic reviews will be the basis of important practice and policy decisions we recommend that the appraisal team agree on how the AMSTAR 2 items should be applied. This includes the practice or policy context and the questions that should be addressed, based on the relevant PICO components. For example, available systematic reviews may have included studies with different comparators or different follow-up times, and their relevance to the policy relevant questions needs to be established. The likely sources of bias should also be agreed on. For instance, in observational studies of intervention effects, confounding by indication (or disease severity) may be problematic when interventions are reserved for certain subgroups of patients.^52^ It is good practice to recruit new users of a technology or drug into studies to avoid prevalence bias.^53^ If the start of one intervention tends to be delayed the choice of comparator may introduce immortal time bias.^54^ Measurement errors can misclassify exposure and outcomes and may be unbalanced across comparison groups. Selective reporting among multiple analyses and outcomes may give an inaccurate measure of intervention effects.

Supplementary appendix 1 provides guidance on sections of AMSTAR 2. Some of the judgments (particularly whether review authors have adequately assessed risk of bias with individual non-randomised studies) are complex, and advice on both methodology and content may be needed. Content knowledge is sometimes necessary to determine if the review authors have made an adequate assessment of the relevant PICO elements (item 1), and to identify potential confounders.

We strongly recommend that individual item ratings are not combined to create an overall score.^55^ ^56^ Rather, users should consider the potential impact of an inadequate rating for each item.

In box 2 we propose a scheme for interpreting weaknesses detected in critical and non-critical items. This is advisory and appraisers should decide which items are most important for the reviews under consideration.

**Box 2 Rating overall confidence in the results of the review**

**High**
*No or one non-critical weakness*: the systematic review provides an accurate and comprehensive summary of the results of the available studies that address the question of interest
**Moderate**
More than one non-critical weakness*: the systematic review has more than one weakness but no critical flaws. It may provide an accurate summary of the results of the available studies that were included in the review
**Low**
*One critical flaw with or without non-critical weaknesses*: the review has a critical flaw and may not provide an accurate and comprehensive summary of the available studies that address the question of interest
**Critically low**
*More than one critical flaw with or without non-critical weaknesses*: the review has more than one critical flaw and should not be relied on to provide an accurate and comprehensive summary of the available studies

*Multiple non-critical weaknesses may diminish confidence in the review and it may be appropriate to move the overall appraisal down from moderate to low confidence

### Inter-rater reliability of AMSTAR 2

We measured inter-rater agreement with three pairs of raters and three sets of systematic reviews (see supplementary appendix 2). The first pair of raters was involved in the development of AMSTAR 2 (coauthors MT and CH). They individually appraised 20 systematic reviews derived from a rapid search (conducted in 2015 on the terms “systematic review” and “meta-analysis” in the title) using Google Scholar. From the first 200 we selected 20 systematic reviews of any healthcare intervention. The other two pairs of raters were experienced in the appraisal of systematic reviews and were not involved in the development of AMSTAR or AMSTAR 2. They applied AMSTAR 2 during their routine work, performing appraisals of systematic reviews of two topics: interventions to reduce medication errors (14 reviews) and non-pharmacological therapies for Parkinson’s disease (20 reviews) (see references in supplementary appendix 2). In both cases systematic reviews had been identified through comprehensive literature searches (details available on request). All raters had access to the user guide (see supplementary appendix 1), applied the instrument individually, and did not try to achieve consensus. In total, six raters applied the instrument to 54 systematic reviews, of which 20 included only randomised controlled trials, 18 included only non-randomised studies of interventions, and 16 included a mixture of both designs.

Supplementary appendix 2 provides summaries of the κ scores for agreement between the three pairs of raters across the three sets of reviews. The values varied substantially across items and between pairs of raters. Most values were in an acceptable range, with 46 of the 50 κ scores falling in the range of moderate or better agreement and 39 displaying good or better agreement. There were no large differences between raters, and those who had been involved in the development of AMSTAR 2 did not have higher levels of agreement than the rater who was not involved. Items 9, 12, and 13 are concerned with measurement of risk of bias and how this is handled during discussion of the meta-analysis and interpretation of the results. The ranges of κ scores for these items were similar to those seen with other items in the instrument (see supplementary appendix 2). For items 9 and 11 the κ values for risk of bias judgments for randomised controlled trials were similar to those for non-randomised studies.

### Usability of AMSTAR 2

The completion times for the 20 reviews used by reviewers 1 and 2 ranged from 15-32 minutes. These estimates do not include the time taken to read the reviews. This is almost twice the time taken to complete the original AMSTAR instrument (range 10-15 minutes), when it was applied to systematic reviews that were limited to randomised controlled trials.^57^ The comments from the reviewers included: that the removal of the “can’t answer” and “not applicable” response options in the original instrument forced them to make judgments; that it takes longer to evaluate the non-randomised and mixed study reviews, but this requires the reviewer to confront important methodological issues; that it was common for review authors to mention the presence or absence of publication bias, but not provide any evidence; and that review authors would disclose their potential competing interests but not how they managed them.

## Discussion

AMSTAR 2 is a major revision of the original AMSTAR instrument, which was designed to appraise systematic reviews that included randomised controlled trials.^22^ ^23^ ^24^ The main modifications include simplified response categories; a more detailed consideration of risk of bias with included studies, and how this was handled by review authors in summarising and interpreting the results of their reviews; better alignment with the PICO framework for research questions; a more detailed justification of selection of study designs for inclusion in a review; and more information on studies that were excluded from reviews. In addition, we recommend defining critical domains before starting an appraisal of a systematic review. Identification of weaknesses in these domains should undermine confidence in the results of a systematic review.

We stress that responses to AMSTAR 2 items should not be used to derive an overall score.^55^ ^56^ The original AMSTAR instrument was often used for this purpose and this was facilitated by the website (www.amstar.ca). We accept that an overall score may disguise critical weaknesses that should diminish confidence in the results of a systematic review and we recommend that users adopt the rating process based on identification of critical domains (see box 2), or some variation based on these principles.^56^

We envisage that AMTAR 2, like its predecessor, may have a role as a convenient teaching aid and as a brief checklist for those conducting systematic reviews. However, we stress that the instrument does not explain in detail the logic and methods of conducting systematic reviews, and those looking for comprehensive advice should consult the Cochrane Handbook.^5^

The consideration of risk of bias in individual studies is equally important for randomised and non-randomised studies of healthcare interventions but is generally better understood with the former. Large non-randomised studies, often conducted in large administrative databases, are increasingly being used to assess the real world impact of a wide range of healthcare technologies and practices. Although such studies often use sophisticated methods, residual confounding or failure to deal with other sources of bias may lead to inaccurate estimates of effect. Inclusion of large observational studies in meta-analyses may generate precise but biased estimates of intervention effects.^32^

The items in AMSTAR 2 that deal with risk of bias identify domains specified in the Cochrane risk of bias instruments for randomised and non-randomised studies.^42^ ^43^ These represent a consensus, in each case developed with input from more than 30 experts in methodology. However, AMSTAR 2 does not currently specify which risk of bias instruments review authors should have used to assess non-randomised studies included in a systematic review. The ROBINS-I instrument, which is the most comprehensive tool for non-randomised studies evaluating the effects of healthcare interventions, was released in 2016 and it is unrealistic to expect authors of reviews started before its release to have used it.^43^ Presently, AMSTAR 2 leaves it to the review authors and those appraising the review to satisfy themselves that the risk of bias instrument used by review authors has sufficient discriminatory ability for the specified risk of bias domains. A review by Sanderson and colleagues identified 86 tools for assessing quality of observational studies, without a clear preference among them.^58^ The authors pointed to the need to agree on critical elements for assessing susceptibility to bias in observational epidemiology. In part this review led to the development of ROBINS-I.^43^ Popular appraisal instruments for individual studies, such as the Newcastle Ottawa Scale and the Scottish Intercollegiate Guidelines Network (SIGN) checklist may not focus on validity alone.^59^ ^60^ The Newcastle Ottawa Scale appears to lack sensitivity and is sometimes used to generate an overall score, something that is not recommended because it may disguise critical weaknesses in a review.^56^ ^61^

AMSTAR 2, as a critical appraisal instrument for systematic reviews, joins several published instruments designed for this purpose.^3^ ^4^ ^16^ ^17^ ^19^ ^20^ ^25^ ^62^ Two prominent examples are concerned with guidelines for reporting systematic reviews, rather than their conduct.^3^ ^4^ Two highly cited instruments were the basis for the development of the original AMSTAR tool.^16^ ^17^ ^22^ Two published instruments are direct derivatives of the original AMSTAR.^19^ ^25^ Another publication includes a checklist used to appraise systematic reviews that are being included in an umbrella review.^20^ Overlap between the content of this checklist and the original AMSTAR is considerable.^22^

AMSTAR 2 provides a broad assessment of quality, including flaws that may have arisen through poor conduct of the review (with uncertain impact on findings). In this respect it differs from another instrument, the Risk Of Bias In Systematic reviews (ROBIS).^62^ ROBIS is a sophisticated three phase instrument that focuses specifically on the risk of bias introduced by the conduct of the review. It covers most types of research question, including diagnosis, prognosis, and aetiology. In contrast, AMSTAR 2 is intended to be used for reviews of healthcare interventions. Inevitably there is overlap in the items considered by ROBIS and AMSTAR 2; indeed, two investigators (BCR, BJS) were involved in the development of both.

In developing AMSTAR 2 we sought to maintain its familiar and popular stepwise checklist approach and augmented this by the addition and modification of items. AMSTAR 2 will be familiar to users of the original instrument, although more demanding to use for reasons discussed previously. Because AMSTAR 2 is structured around the key sequential steps in the conduct of a systematic review, it may be used as a brief teaching aid or as a checklist by those conducting systematic reviews.

Unlike the original instrument, AMSTAR 2 identifies critical weaknesses (see box 1) that should reduce confidence in the findings of a review, and it asks users to prespecify how this list will vary for the review topic. We understand that there will be debate about membership of this list and propose that users may wish to prespecify a different set of critical items for a specific PICO research question or setting.

We did not perform an extensive validation of the revised AMSTAR 2 tool. In its development, 10 domains were retained from the original validated tool, albeit with some wording changes based on feedback and extensive experience of using it. Two domains were given more detailed coverage: duplicate study selection and data extraction now have their own items (they were combined in the original tool); we have added more detailed, and separate, considerations of risk of bias for randomised and non-randomised studies. The sub-items were derived from widely used Cochrane instruments. One domain was removed; consideration of grey literature, previously a separate item, is now handled in the item on literature searching. In total, four domains were added. Two of these come directly from the ROBINS-I tool—namely, elaboration of PICO in the review and the way in which risk of bias was handled during evidence synthesis.^43^ One of the other new domains, discussion of possible causes and importance of heterogeneity, is elaboration of content in the original AMSTAR tool.^22^ The final domain, justification of selection of study designs, is justified by adapting AMSTAR to deal with non-randomised designs. We do not think this needs validation because we believe it is obvious that authors of systematic reviews should justify why they have included study designs that are more susceptible to bias.

The levels of agreement achieved by the three pairs of raters varied across items, but they were moderate to substantial for most items. Notably, the agreement between two raters involved in the development of AMSTAR 2 was no higher than that achieved by experienced raters who had not been involved its development. We did not expect perfect agreement, and differences between raters reflect the demanding nature of some item level judgments and should prompt group discussion of their causes and importance, and, if needed, consultation with experts in subject matter and methods.

In developing AMSTAR 2 we relied heavily on the consensus of the expert panel, but we also received extensive feedback from users of the original instrument in the form of direct communications, website comments, and evaluations made at teaching workshops at Cochrane Colloquiums. In the later phases of development of AMSTAR 2 we had access to, and discussed, recently published critiques of AMSTAR.^25^ ^26^ ^27^ ^28^ ^29^ ^30^ ^31^

Our experience of releasing and using the original AMSTAR instrument is that judgments need to be made and users may sometimes decide to make modifications to the instrument.^25^ ^26^ ^30^ We encourage investigators to provide feedback, and, if they adapt the instrument for particular settings, to report their experience at www.amstar.ca.

## Web extra

Extra material supplied by the authors

**Supplementary figure:** the AMSTAR 2 instrument

**Supplementary appendix 1:** AMSTAR 2 guidance document

**Supplementary appendix 2:** Inter-rater reliability of AMSTAR 2 items and references for studies included in the analyses

## Notes

Provenance and peer review: Not commissioned; externally peer reviewed.
