# Supplementary material for: AMSTAR 2: a critical appraisal tool for systematic reviews that include randomised or non-randomised studies of healthcare interventions, or both
Source: BMJ. 2017 Sep 21;358:j4008. doi: 10.1136/bmj.j4008 (PMC5833365; doi:10.1136/bmj.j4008)
Supplement: Supplementary file 3 — Supplementary appendix 2: Inter-rater reliability of AMSTAR 2 items and references for studies included in the analyses [file sheb036104.ww2.pdf]

Online Appendix 2: Inter-rater reliability of AMSTAR 2 items and references for studies included in the analyses

	**Raters 1 and 2** (20 reviews: studies of multiple interventions and associations) Supplementary references 1-20			**Raters 3 and 4** (14 reviews: studies of interventions to improve medication safety) Supplementary references 21-34			**Raters 5 and 6** (20 reviews: studies of therapies in Parkinson's Disease) Supplementary references 35-54		
**Item**	**Cohen Kappa**	**95% confidence interval**	**Observed (O) and Expected (E) Agreement (%)**	**Cohen Kappa**	**95% confidence interval**	**Observed (O) and Expected (E) Agreement (%)**	**Cohen Kappa**	**95% confidence interval**	**Observed (O) and Expected (E) Agreement (%)**
1	0.69	0.37, 1.0	O 85.7<br>E 54.0	#	**	O 100<br>E 100	0.86	0.60, 1.0	O 95<br>E 64
2	0.38	0.06, 0.81;	O 92.9<br>E 88.6	1.0	1.0, 1.0	O 92.9<br>E 48.2	0.64	0.01, 1.0	O 95<br>E 86
3	0.39	0.05, 0.83;	O 76.2<br>E 61.2	0	**	O 92.9<br>E 92.9	0.71	0.34, 1.0	O 90<br>E 65.5
4	0.61	0.30, 0.93;	O 81<br>E 51	0.31	-0.11, 0.73	O 71.4<br>E 58.7	0.77	0.47, 1.0	O 95<br>E 78
5	0.72	0.44, 1.0;	O 85.7<br>E 49.0	0.85	0.57, 1.0	O 92.9<br>E 52	0.69	0.41, 0.98	O 80<br>E 35
6	0.63	0.32, 0.93;	O 81.0<br>E 49.2	0.71	0.33, 1.0	O 85.7<br>E 51	0.80	0.53, 1.0	O 90<br>E 50.5
7	0.34	-0.13, 0.81;	O 78.6<br>E 67.5	0.76	0.31, 1.0	O 92.9<br>E 70.4	0.73	0.44, 1.0	O 90<br>E 61.8
8	0.78	0.51, 1.0;	O 95.2<br>E 78.2	0.75	0.47, 1.0	O 92.9<br>E 71.4	0.51	0.22, 0.81	O 70<br>E 38.3
9 (RCT)	0.48	0.22, 0.73;	O 82.5<br>E 66.7	0.90	0.69, 1.0	O 96.4<br>E 65.8	0.47	0.15, 0.79	O 80<br>E 62
9 (NRSI)	0.63	0.25, 1.0;	O 90.5<br>E 74.5	0.81	0.55, 1.0	O 92.9<br>E 62.8	0.52	0.30, 0.75	O 75<br>E 47.5
10	0	**	O 95.2<br>E 95.2	0	**	O 85.7<br>E 85.7	0.81	0.52, 1.0	O 95<br>E 73
11 (RCT)	0.72	0.44, 1.0;	O 90.5<br>E 66.0	0.86	0.59, 1.0	O 92.9<br>E 50	0.89	0.71, 1.0	O 95<br>E 52.8
11 (NRSI)	0.92	0.79, 1.0	O 98.4<br>E 78.8	0.72	0.26, 1.0	O 85.7<br>E 49	0.72	0.46. 0.98	O 85<br>E 45.8

Online Appendix 2: Inter-rater reliability of AMSTAR 2 items and references for studies included in the analyses

12	0.43	0.01, 0.84	O 78.6<br>E 62.5	1.0	1.0, 1.0	O 100<br>E 40	0.71	0.45, 0.97	O 85<br>E 48.8
13	0.71	0.45, 0.98	O 85.7<br>E 50.6	0.85	0.56, 1.0	O 92.3<br>E 49.7	0.59	0.23, 0.94	O 80<br>E 51.5
14	0.90	0.70, 1.0	O 95.2<br>E 54	0.85	0.57, 1.0	O 92.9<br>E 52	0.77	0.52, 1.0	O 92.5<br>E 67
15	0.63	0.25, 1.0	O 85.7<br>E 61.2	1.0	1.0, 1.0	O 100<br>E 75.5	0.62	0.15, 1.0	O 90<br>E 74
16	0.83	0.52, 1.0	O 95.2<br>E 71.4	0.59	0.11, 1.0	O 85.7<br>E 66.3	0.76	0.45, 1.0	O 90<br>E 58

Not calculated – division by zero

** Confidence interval not calculated

Supplementary references used in assessing inter-rater agreement

Systematic reviews appraised by raters 1 and 2 (mixture of interventions and settings)

1. Ansaloni L, Catena F, Coccolini F, Ercolani G, Gazzotti F, Pasqualini E, Pinna AD. Surgery versus conservative antibiotic treatment in acute appendicitis: a systematic review and meta-analysis of randomized controlled trials. Digestive surgery. 2011 May 3;28(3):210-21.
2. Barron SJ, Del Vecchio MT, Aronoff SC. Intravenous immunoglobulin in the treatment of Stevens–Johnson syndrome and toxic epidermal necrolysis: a meta-analysis with meta-regression of observational studies. International journal of dermatology. 2015 Jan 1;54(1):108-15.
3. Brown C, Subramanian V, Wilcox CM, Peter S. Somatostatin analogues in the treatment of recurrent bleeding from gastrointestinal vascular malformations: an overview and systematic review of prospective observational studies. Digestive diseases and sciences. 2010 Aug 1;55(8):2129-34.
4. Forst T, Hanefeld M, Jacob S, Moeser G, Schwenk G, Pfützner A, Haupt A. Association of sulphonylurea treatment with all-cause and cardiovascular mortality: A systematic review and meta-analysis of observational studies. Diabetes and Vascular Disease Research. 2013 Jan 4:1479164112465442.
5. Hongliang Y, Yin L, Jiang X, Sun X, Wu J, Tian H, Gao X, He X. Effect of metformin on cancer risk and treatment outcome of prostate cancer: a meta-analysis of epidemiological observational studies. PloS one. 2014 Dec 29;9(12):e116327.

6. Hsu J, Santesso N, Mustafa R, Brozek J, Chen YL, Hopkins JP, Cheung A, Hovhannisyan G, Ivanova L, Flottorp SA, SÃĶterdal I. Antivirals for treatment of influenza: a systematic review and meta-analysis of observational studies. Annals of internal medicine. 2012 Apr 3;156(7):512-24.
7. Kamel H, Navi BB, Nakagawa K, Hemphill III JC, Ko NU. Hypertonic saline versus mannitol for the treatment of elevated intracranial pressure: A meta-analysis of randomized clinical trials*. Critical care medicine. 2011 Mar 1;39(3):554-9.
8. Klatte T, Grubmüller B, Waldert M, Weibl P, Remzi M. Laparoscopic cryoablation versus partial nephrectomy for the treatment of small renal masses: systematic review and cumulative analysis of observational studies. European Urology. 2011 Sep 30;60(3):435-43.
9. Kovacs FM, Urrútia G, Alarcón JD. Surgery versus conservative treatment for symptomatic lumbar spinal stenosis: a systematic review of randomized controlled trials. Spine. 2011 Sep 15;36(20):E1335-51.
10. Larsson ME, Käll I, Nilsson-Helander K. Treatment of patellar tendinopathy—a systematic review of randomized controlled trials. Knee surgery, sports traumatology, arthroscopy. 2012 Aug 1;20(8):1632-46.
11. Liu J, Chen Q. Effect of statins treatment for patients with aneurysmal subarachnoid hemorrhage: a systematic review and meta-analysis of observational studies and randomized controlled trials. International journal of clinical and experimental medicine. 2015;8(5):7198.
12. Matlaga BR, Jansen JP, Meckley LM, Byrne TW, Lingeman JE. Treatment of ureteral and renal stones: a systematic review and meta-analysis of randomized, controlled trials. The Journal of urology. 2012 Jul 31;188(1):130-7.
13. Musso G, Gambino R, Cassader M, Pagano G. A meta-analysis of randomized trials for the treatment of nonalcoholic fatty liver disease. Hepatology. 2010 Jul 1;52(1):79-104.
14. Petersen LK, Christensen K, Kragstrup J. Lipid-lowering treatment to the end? A review of observational studies and RCTs on cholesterol and mortality in 80+-year olds. Age and ageing. 2010 Nov 1;39(6):674-80.
15. Reich K, Burden AD, Eaton JN, Hawkins NS. Efficacy of biologics in the treatment of moderate to severe psoriasis: a network meta-analysis of randomized controlled trials. British Journal of Dermatology. 2012 Jan 1;166(1):179-88.
16. Vasiliadis HS, Wasiak J, Salanti G. Autologous chondrocyte implantation for the treatment of cartilage lesions of the knee: a systematic review of randomized studies. Knee surgery, sports traumatology, arthroscopy. 2010 Dec 1;18(12):1645-55.
17. Vieta E, Locklear J, Günther O, Ekman M, Miltenburger C, Chatterton ML, Åström M, Paulsson B. Treatment options for bipolar depression: a systematic review of randomized, controlled trials. Journal of clinical psychopharmacology. 2010 Oct 1;30(5):579-90.
18. Wang CH, Ma MH, Chou HC, Yen ZS, Yang CW, Fang CC, Chen SC. High-dose vs non–high-dose proton pump inhibitors after endoscopic treatment in patients with bleeding peptic ulcer: a systematic review and meta-analysis of randomized controlled trials. Archives of internal medicine. 2010 May 10;170(9):751-8.

19. Welsh T, Gladman J, Gordon AL. The treatment of hypertension in care home residents: a systematic review of observational studies. Journal of the American Medical Directors Association. 2014 Jan 31;15(1):8-16.
20. Zheng Z, Liang W, Milgrom DP, Zheng Z, Schroder PM, Kong NS, Yang C, Guo Z, He X. Liver transplantation versus liver resection in the treatment of hepatocellular carcinoma: a meta-analysis of observational studies. Transplantation. 2014 Jan 27;97(2):227-34.

Systematic reviews appraised by raters 3 and 4 (interventions to improve medication safety)

21. Acheampong F, Anto BP, Koffuor GA. Medication safety strategies in hospitals–a systematic review. International Journal of Risk & Safety in Medicine. 2014 Jan 1;26(3):117-31.
22. Boeker EB, de Boer M, Kiewiet JJ, Lie-A-Huen L, Dijkgraaf MG, Boermeester MA. Occurrence and preventability of adverse drug events in surgical patients: a systematic review of literature. BMC health services research. 2013 Sep 28;13(1):364.
23. Boeker EB, Ram K, Klopotowska JE, Boer M, Creus MT, Andrés AL, Sakuma M, Morimoto T, Boermeester MA, Dijkgraaf MG. An individual patient data meta-analysis on factors associated with adverse drug events in surgical and non-surgical inpatients. British journal of clinical pharmacology. 2015 Apr 1;79(4):548-57.
24. Damiani G, Pinnarelli L, Scopelliti L, Sommella L, Ricciardi W. A review on the impact of systematic safety processes for the control of error in medicine. Medical Science Monitor. 2009 Jun 23;15(7):RA157-66.
25. Hakkarainen KM, Hedna K, Petzold M, Hägg S. Percentage of patients with preventable adverse drug reactions and preventability of adverse drug reactions–a meta-analysis. PloS one. 2012 Mar 15;7(3):e33236.
26. Hodgkinson B, Koch S, Nay R, Nichols K. Strategies to reduce medication errors with reference to older adults. International Journal of Evidence-Based Healthcare. 2006 Mar 1;4(1):2-41.
27. Kanagaratnam L, Dramé M, Trenque T, Oubaya N, Nazeyrollas P, Novella JL, Jolly D, Mahmoudi R. Adverse drug reactions in elderly patients with cognitive disorders: A systematic review. Maturitas. 2016 Mar 31;85:56-63.
28. Maaskant JM, Vermeulen H, Apampa B, Fernando B, Ghaleb MA, Neubert A, Thayyil S, Soe A. Interventions for reducing medication errors in children in hospital. The Cochrane Library. 2015 Jan 1.
29. Manias E, Kinney S, Cranswick N, Williams A, Borrott N. Interventions to reduce medication errors in pediatric intensive care. Annals of Pharmacotherapy. 2014 Oct;48(10):1313-31.

30. Manias E, Williams A, Liew D. Interventions to reduce medication errors in adult intensive care: a systematic review. British journal of clinical pharmacology. 2012 Sep 1;74(3):411-23.
31. Marasinghe KM. Computerised clinical decision support systems to improve medication safety in long-term care homes: a systematic review. BMJ open. 2015 May 1;5(5):e006539.
32. Nuckols TK, Smith-Spangler C, Morton SC, Asch SM, Patel VM, Anderson LJ, Deichsel EL, Shekelle PG. The effectiveness of computerized order entry at reducing preventable adverse drug events and medication errors in hospital settings: a systematic review and meta-analysis. Systematic reviews. 2014 Jun 4;3(1):56.
33. Salmasi S, Khan TM, Hong YH, Ming LC, Wong TW. Medication errors in the Southeast Asian countries: a systematic review. PLoS One. 2015 Sep 4;10(9):e0136545.
34. Wang T, Benedict N, Olsen KM, Luan R, Zhu X, Zhou N, Tang H, Yan Y, Peng Y, Shi L. Effect of critical care pharmacist's intervention on medication errors: a systematic review and meta-analysis of observational studies. Journal of critical care. 2015 Oct 31;30(5):1101-6.

Systematic reviews appraised by raters 5 and 6 (therapies for Parkinson's Disease)

35. Aguiar LPC, da Rocha PA, Morris M. Therapeutic Dancing for Parkinson's Disease. International Journal of Gerontology 2016;10(2):64-70.
36. Allen NE, Sherrington C, Paul SS, Canning CG. Balance and falls in Parkinson's disease: A meta-analysis of the effect of exercise and motor training. Movement disorders 2011;26(9):1605-15.
37. Aman JE, Elangovan N, Yeh IL, Konczak J+. The effectiveness of proprioceptive training for improving motor function: a systematic review. Frontiers in human neuroscience 2014;8.
38. Cai JP, Chen WJ, Lin Y, Cai B, Wang N. Safety and efficacy of rasagiline in addition to levodopa for the treatment of idiopathic Parkinson's disease: a meta-analysis of randomised controlled trials. European neurology 2015;73(1-2):5-12.
39. Fok P, Farrell M, McMeeken J, Kuo YL. The effects of verbal instructions on gait in people with Parkinson's disease: a systematic review of randomized and non-randomized trials. Clinical rehabilitation 2011;25(5):396-407.
40. Hart RG, Pearce LA, Ravina BM, Yaltho TC, Marler JR. Neuroprotection trials in Parkinson's disease: systematic review. Movement disorders 2009;24(5):647-54.
41. Hayes HA, Hunsaker N, Dibble LE. Implicit motor sequence learning in individuals with parkinson disease: a meta-analysis. Journal of Parkinson's Disease 2015;549-60.

42. Hawkey NM, Zaorsky NG, Galloway TJ. The role of radiation therapy in the management of sialorrhea: A systematic review. The Laryngoscope 2016;126(1):80-5.
43. Kwakkel G, De Goede CJT, Van Wegen EEH. Impact of physical therapy for Parkinson's disease: a critical review of the literature. Parkinsonism & related disorders 2007;13:S478-S487.Lau RW, Teo T, Yu F, Chung RC, Pang MY. Effects of whole-body vibration on sensorimotor performance in people with Parkinson disease: a systematic review. Physical therapy 2011;91(2):198.
44. Lam YC, Kum WF, Durairajan SSK, Lu JH, Man SC, Xu M, et al. Efficacy and safety of acupuncture for idiopathic Parkinson's disease: a systematic review. The Journal of Alternative and Complementary Medicine 2008;14(6):663-71.
45. Lau RWK, Teo T, Yu F, Chung RCK, Pang MYC. Effects of whole-body vibration on sensorimotor performance in people with Parkinson Disease: A systematic review. Physical Therapy 2011; 91(2): 198-209.
46. Lee MS, Lam P, Ernst E. Effectiveness of tai chi for Parkinson's disease: a critical review. Parkinsonism & related disorders 2008;14(8):589-94.
47. Lee MS, Shin B, Kong JC, Ernst E. Effectiveness of acupuncture for Parkinson's disease: A systematic review. Movement disorders 2008;23(11):1505-15.
48. Liu J, Wang Ln. Mitochondrial enhancement for neurodegenerative movement disorders: a systematic review of trials involving creatine, coenzyme Q10, idebenone and mitoquinone. CNS drugs 2014;28(1):63-8.
49. Murray DK, Sacheli MA, Eng JJ, Stoessl AJ. The effects of exercise on cognition in Parkinson's disease: a systematic review. Translational neurodegeneration 2014;3(1):5.
50. Ni X, Liu S, Lu F, Shi X, Guo X. Efficacy and safety of Tai Chi for Parkinson's disease: a systematic review and meta-analysis of randomized controlled trials. PLoS One 2014;9(6):e99377.
51. Sharififar S, Coronado RA, Romero S, Azari H, Thigpen M. The effects of whole body vibration on mobility and balance in Parkinson disease: a systematic review. Iranian journal of medical sciences 2014;39(4):318-26.
52. Shen X, Wong-Yu IS, Mak MK. Effects of exercise on falls, balance, and gait ability in Parkinson's disease: a meta-analysis. Neurorehabilitation and neural repair 2016;30(6):512-27.
53. Toh SFM. A systematic review on the effectiveness of Tai Chi exercise in individuals with Parkinson's disease from 2003 to 2013. Hong Kong Journal of Occupational Therapy 2013;23(2):69-81.
54. Wunderer K, Schabrun SM, Chipchase LS. The effect of whole body vibration in common neurological conditions-a systematic review. Physical Therapy Reviews 2008;13(6):434-42.

Online Appendix 2: Inter-rater reliability of AMSTAR 2 items and references for studies included in the analyses
